# Ultra-/Small Angle X-ray Scattering (USAXS/SAXS) and Static Light Scattering (SLS) Modeling as a Tool to Determine Structural Changes and Effect on Growth in *S. epidermidis*

**DOI:** 10.1021/acsabm.2c00218

**Published:** 2022-07-29

**Authors:** Hugo Duarte, Jeremie Gummel, Eric Robles, Debora Berti, Emiliano Fratini

**Affiliations:** † Department of Chemistry “Ugo Schiff” and CSGI, University of Florence, Sesto Fiorentino, Florence I-50019, Italy; ‡ Brussels Innovation Centre, Temselaan 100, Strombeek-bever B-1853, Belgium; § Household Care Analytical, Procter & Gamble Newcastle Innovation Centre, Newcastle NE12 9TS, United Kingdom

**Keywords:** small angle scattering, USAXS, SAXS, static light scattering, bacteria, antimicrobial, modeling

## Abstract

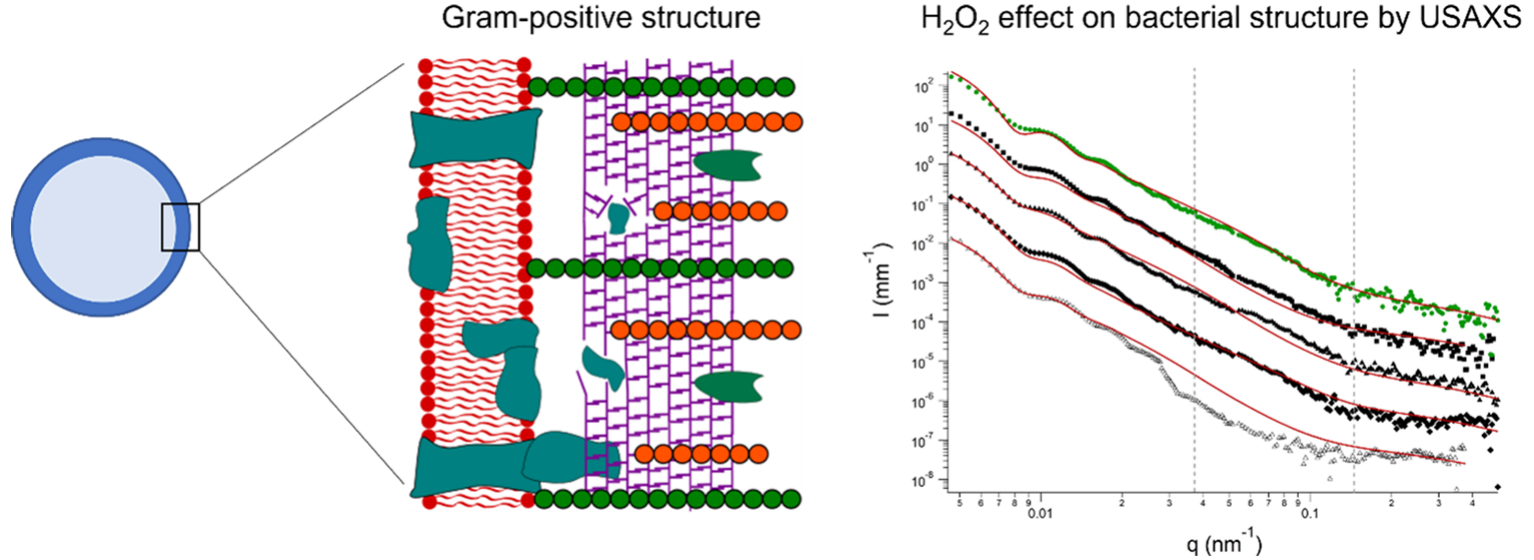

Usually, to characterize bacterial cells’ susceptibility to antimicrobials, basic microbiology techniques such as serial dilutions or disk assays are used. In this work, we present an approach focused on combining static light scattering (SLS) and ultra-/small angle X-ray scattering (USAXS/SAXS). This approach was used to support microbiology techniques, with the aim of understanding the structural changes caused to bacteria when they are exposed to different stresses like pH, oxidation, and surfactants. Using USAXS/SAXS and SLS data, we developed a detailed multiscale model for a Gram-positive bacterium, *S. epidermidis*, and we extracted information regarding changes in the overall size and cell thickness induced by different stresses (i.e., pH and hydrogen peroxide). Increasing the concentration of hydrogen peroxide leads to a progressive reduction in cell wall thickness. Moreover, the concomitant use of pH and hydrogen peroxide provides evidence for a synergy in inhibiting the *S. epidermidis* growth. These promising results will be used as a starting base to further investigate more complex formulations and improve/refine the data modeling of bacteria in the small angle scattering regime.

## 1 Introduction

**Figure 1:**
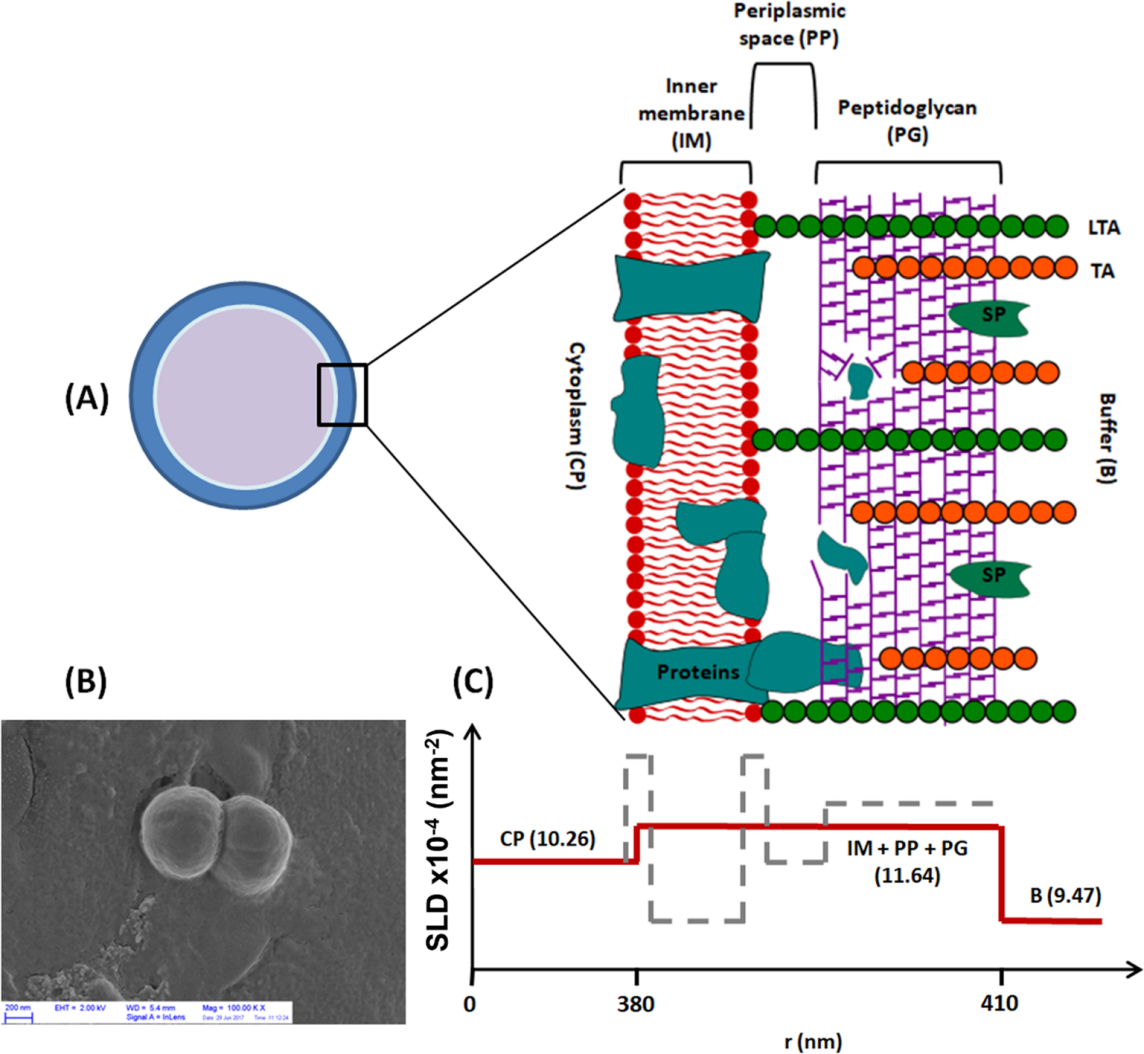
(A) Schematic representation of the cross section of a bacterial cell in the case of a Gram-positive cell wall. Similarly, to the Gram-negative scheme:^12^ the cell cytoplasm is separated from the periplasm (PP) by the inner membrane (IM) composed by lipopolysaccharides and proteins where lipoteichoic acids (LPA) are bound, protruding through the peptidoglycan (PG) layer. On Gram-positive cells, the wall is mainly composed by a thick PG layer with attached surface proteins (SP) and teichoic acids (TA). (B) SEM micrograph of a *S. epidermidis* cell (during division). (C) Scattering length density profile for a Gram-positive membrane (dashed gray line) and simplified version of the same profile (continuous red line) used in the core–shell model for X-rays (see eq 1).

Due to the progressively increasing resistance of bacteria toward antibiotics, the research for different approaches and alternative therapies to suppress bacterial growth in different cases is a top priority.^1^ Bacterial cells need to maintain their membrane architecture for regulating the trans-membrane potential, an essential requisite for growth as well as metabolic activity.^2^ The Gram-positive cell wall is composed of a thick peptidoglycan layer, presenting a greater variation in the composition and structural arrangement when compared with Gram-negative species.^3^ The peptidoglycan main function is to preserve cell integrity, resisting turgor, and any inhibition of its biosynthesis or degradation during cell growth will result in cell lysis.^4^ The cell wall can be disrupted, for example, by cationic antimicrobial agents, and consequent changes in bacteria size/shape, cell wall thickness, or even proteins and DNA/RNA 3D structure can be accessed by small angle scattering techniques.^2,5−7^ Some of the initial approaches for conducting light scattering experiments on bacteria were reported by P. J. Wyatt.^8−10^ These studies were based on the mathematical assumption that “each distinct organism that scatters light is an essentially unique scattering pattern”, as a natural consequence of the unique structural and biochemical features that distinguish one microorganism from another. In other words, if all aspects of the light scattering characteristics of a particular microorganism could be measured, its structure and spatial biochemical composition could in principle also be determined. Though the overall biochemical constituents of the various bacterial features are well-known, little is known about their spatial distribution. It should also be kept in mind that if a microorganism is to be identified through its scattering pattern at a fixed wavelength of light, the smallest structural differences that may be detected will be in the range of an order of magnitude lower than the scattering wavelength.^9^ Koch in 1961 showed that it is possible to apply the Rayleigh–Gans theory for the study of light scattered by proteins and small colloidal particles as well as to objects as large as bacteria and mitochondria with some discrepancies at high angles between theory and experiments, due to the high refraction index of the cell walls or from the irregular distribution of cell materials. As for these types of biological materials, most of the light is scattered in the forward direction; low angle scattering measurements would provide a quite accurate average particle volume.^11^ More recently through small angle X-ray scattering (SAXS) and small angle neutron scattering (SANS), a very detailed model for the ultrastructure of *E. coli* has been proposed.^12^ By combining SAXS and SANS, the overall geometry of the whole body and the details of the cell envelope at the micro/nanoscale were elucidated. Comparing the scattering patterns coming from both probes allowed the exclusion of X-ray radiation damage from X-rays and toxic effects due to the deuterated medium. The main achievement was the formulation of a global model by combining the formalisms of a colloidal core–shell to describe the cell body, a lipid-membrane for the cell envelope, and a polymer-chain to describe the flagella. In the end, Semeraro et al.^12^ also proposed that the approach taken may be applied to probe the *in vivo* effect of detergents, antibiotics, and antimicrobial peptides on the bacterial cell wall. In agreement with this statement, in the present work we aim to demonstrate how USAXS/SAXS and light scattering may be used with such a goal directly in the treating medium without any pretreatment. In our case, a different model for the bacterial cell ultrastructure was considered as we worked with Gram-positive bacteria (see Figure 1 for further details). Moreover, we intend to correlate data obtained from the synchrotron source with the ones obtained by light scattering to identify the effect of antimicrobial compounds on the structure of bacteria in terms of changes in the overall size and wall thickness.^8,12^ This approach might be useful and complementary in any field that uses standard microbiology techniques such as inhibition ring assay, colony density, and bacteriostatic and/or bactericidal tests, and it requires a deeper understanding of the effects of antibacterial solutions on the microorganism structure as it could identify synergies between components and provide clues regarding the mechanism of action of the compounds of interest.^7,13,22,14−21^

## 2 Materials and Methods

### 2.1 Chemicals and Growth Media

Tryptic soy broth (TSB), tryptic soy agar (TSA), Tween 20, saline solution (0.85 wt %), and hydrogen peroxide were obtained from Sigma-Aldrich and used as received. Phosphate buffer solution (PBS) was prepared *in situ*, and the pH was adjusted with hydrochloric acid or sodium hydroxide to the chosen value.

### 2.2 Bacterial Strains and Culturing Conditions

Gram-positive *Staphylococcus epidermidis* (NCTC 11047) was chosen as the model bacterium, representing one of the emerging multiresistant species. It possesses a biosafety level 1, a spherical shape making it an attractive microorganism to be modeled from a structural point of view while retaining important features related to more dangerous *Staphylococcus aureus* or other Gram-positive bacteria. Growth curves were obtained from cells grown in TSB in 10 mL tubes with pressure caps (not sealed so air could circulate) agitated for 24 h at 180 rpm and 37 °C in an incubator (see Supporting Information for details).

### 2.3 Modeling Bacterial Growth: Modified Gompertz Model

As a first step, *S. epidermidis* cells’ growth on TSB was assessed (Figures S1 and S2). The growth curve shown is a result of a set of experiments where three different inoculum concentrations were tested with good reproducibility and accuracy, as in the lag phase the measured optical density at 600 nm was 0.317 ± 0.002. After this, optical density values were correlated with the colony forming units per volume (CFU/mL) by the serial dilution method.^23,24^ While cells were growing, aliquots were taken at certain times, and a series of dilutions were prepared and then plated (method detailed in SI).

### 2.4 Sample Preparation for USAXS/SAXS and Light Scattering

For USAXS measurements, cells were grown in TSA for 24 h at 37 °C, resuspended in saline solution (0.85 wt %) and then mixed with TSB (3 wt %). For light scattering, sample preparation was similar so as to correlate with X-ray data, where the cell suspension concentration was set to 10^8^ CFU/mL and diluted 1:2 for the experiments, having in the end 5 × 10^7^ CFU/mL. Cells were also grown in TSA, for the same time and at the same temperature, and washed with saline solution, and the optical density was measured to obtain 10^9^ CFU/mL. The suspension was then diluted 1:10, mixed with the PBS or PBS and hydrogen peroxide mixture, and incubated for 1 h. After this time, the suspensions were washed twice by cycles of centrifugation (5000 rpm) and suspension in distilled water. In the end, a bacterial suspension of 10^8^ CFU/mL was diluted 1:10, and 10^7^ CFU/mL was the concentration chosen for all experiments. The explanation for choosing such a concentration is detailed in the Results and Discussion section.

### 2.5 USAXS/SAXS

USAXS/SAXS was performed on the ID02 beamline at the European Synchrotron Radiation Facility (ESRF), in Grenoble, France. A wavelength of 0.1 nm and a sample-to-detector distance of 15 m were used to cover a *q*-range of 0.005–0.5 nm^–1^ where *q* is the scattering vector: *q* = (4π/λ) sin(θ/2), with λ the wavelength and θ the scattering angle. The diameter of the capillary was 2 mm. The 2D SAXS pattern was collected by using a Rayonix MX170 CCD detector. To maximize the signal/noise ratio due to the inherent large dynamic range in the bacteria scattering, we recorded for each condition first a 1 × 1 binned pattern with a standard beam-stop and second a 4 × 4 binned pattern with a large beam-stop to enhance the high-*q* signal without saturating the detector. After the data reduction, both *I* vs *q* curves were merged to create the scattering patterns used for data fitting and interpretation. Measured scattering intensities were normalized to absolute scale (d*∑*/d*Ω*) and background corrected by standard procedures implemented at ESRF.^25^

As described by Semeraro et al.^12^ for a Gram-negative strain, the scattering length density for each section of the cell wall can be considered. Gram-positive bacteria developed a different cell-envelope structure than Gram-negative cells; lacking the outer membrane, the cell wall is usually much thicker with multiple peptidoglycan layers. Gram-positive membranes are also very variable, possessing a constant motif, glycol-polymers that form part of the fabric of the cell, attached either to the peptidoglycan (teichoic acids) or to the membrane lipids (lipoteichoic acids).^26^ A sketch of the structure of a Gram-positive bacterium is reported in Figure 1A with the fine details of the cell wall. Figure 1B shows an SEM micrograph to confirm the almost spherical structure of the *S. epidermidis*. Figure 1C reports the SLD profile obtained considering all the cellular layers (Figure 1C, dashed gray line) and corresponding to a multishell model. Though this model could be considered for the most accurate representation, a simplified core–shell model was chosen in our approach due to the lack of neutron scattering experiments. The SLD profile also had to be adapted, thus considering the wall with one layer composed by the inner membrane, periplasmic space, and the peptidoglycan (Figure 1C, continuous red line). Parameters to calculate the SLD profile were taken from the literature,^12^ and small adjustments were made with regard to the cell core and the peptidoglycan layer as we worked with a different strain than the one presented by Semeraro et al.^12^ (see Table S1). The SLD value for the cytoplasm was obtained, allowing the parameter to vary during the fitting of the pristine bacterial dispersion.

The SLD value for the peptidoglycan layer of *S. epidermidis* cells was calculated taking into account the constitution of the polymer.^3^ In particular, the *S. epidermidis* peptidoglycan is generally composed of lysine, glycine, serine, *N*-acetylmuramic acid (MurNac), and *N*-acetylglucosamine (NAG) in different proportions (0.25; 1; 0.25; 0.25; 0.25; respectively). We also considered the hydration effect, and the same calculations were performed for *E. coli* cells for comparison with Semeraro et al.^12^

Data analysis was performed with SasView software.^27^ The model used to fit USAXS/SAXS curves is the result of the sum of two contributions *I*(*q*) = *I*_cs_(*q*) + *I*_gc_(*q*), in an effort to accurately describe the *S. epidermidis* cell ultrastructure. The first contribution is a core–shell form factor, *I*_cs_(*q*). It dominates the scattering at low *q* values, from 5 × 10^–3^ to 0.15 × 10^–1^ nm^–1^, and describes the cell shape and wall structure:
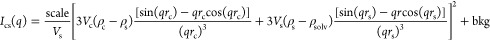
1Here, scale is a scale factor, *V*_s_ is the volume of the cell wall, *V*_c_ is the volume of the cell core (i.e., the cytoplasmic region), *r*_s_ is the radius of the cell wall, *r*_c_ is the radius of the cytoplasm, ρ_c_ is the scattering length density (SLD) of the cytoplasm, ρ_s_ is the SLD of the wall, ρ_solv_ is the SLD of the buffer, and bkg is the background. The SLD profile associated with the cell in the case of X-ray radiation is shown in Figure 1C. Equation 1 has been also extended to consider the polydispersity on *r*_c_ and on the thickness of the membrane (*r*_s_*– r*_c_).

In addition to this core–shell form factor, we then considered a polydisperse Gaussian coil function, *I*_gc_(*q*), to describe the scattering from the inner cytoplasm content as DNA and proteins, which are contributing to the data at high *q* values (up to 0.45 × 10^–1^ nm^–1^):
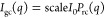
2where
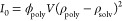
3
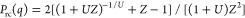
4
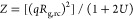
5
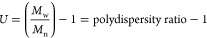
6
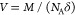
7ϕ_poly_ is the volume fraction of the cytoplasm material, *V* is the volume of a polymer coil, which here is an average volume of cytoplasm material, *M*_w_ and *M*_n_ are, respectively, the number- and weight-average molecular weights of the biopolymer mixture (considering it as DNA and proteins), *N*_A_ is Avogadro’s number, δ is the bulk density of the cytoplasm material, ρ_poly_ is the SLD of the of the cytoplasm constituents, ρ_solv_ is the SLD of the buffer, and *R*_g*,*rc_ is the average radius of gyration of the cytoplasm constituents. Further details on the models can be found in the SasView documentation.^27^

### 2.6 Light Scattering

The light scattering profile was obtained from an LS apparatus (LS instruments) equipped with a 638 nm laser and a goniometer arm able to move continuously from 20° to 150°. The *q* range probed in SLS experiments is smaller (from 4 × 10^–3^ to 5 × 10^–2^ nm^–1^) than the one probed by X-rays, corresponding only to the region modeled as a core–shell. For this reason, data analysis was performed with SasView software,^27^ applying only the core–shell model (see eq 1) and considering the appropriate SLD in the case of light (i.e., SLD values for X-rays are replaced by the corresponding refractive indexes, *n*). According to the literature,^8−10^ the refractive index for the cellular cytoplasm (*n*_c_) is about 1.365, while it increases to 1.42 for the cell wall (*n*_s_) and results in 1.33 when considering the solvent (*n*_solv_).

### 2.7 SEM

After exposure to certain conditions, a 20 μL drop of bacteria suspension was cast on a sample holder, vacuum-dried, and gold-coated. Imaging was performed by means of a ∑IGMA field emission SEM (Carl Zeiss Microscopy GmbH, Germany).

### 2.8 Fluorescence Microscopy

Fluorescence micrographs were taken from *S. epidermidis* cells with a Leica DFC450C. Cells were dyed with the L7007 BacLight live/dead assay from Thermo Fisher. The protocol consists of the mixture of equal volumes of two dyes, Syto 9 and propidium iodide, after washing cells with an adequate buffer. The mixture is incubated at room temperature in the dark for 15 min, and samples are ready for imaging.

### 2.9 Optical Microscopy

A Nikon Diaphot inverted microscope equipped with a Nikon CCD camera was used for optical microscopy.

## 3 Results and Discussion

### 3.1 *S. epidermidis* at pH 7

**Figure 2:**
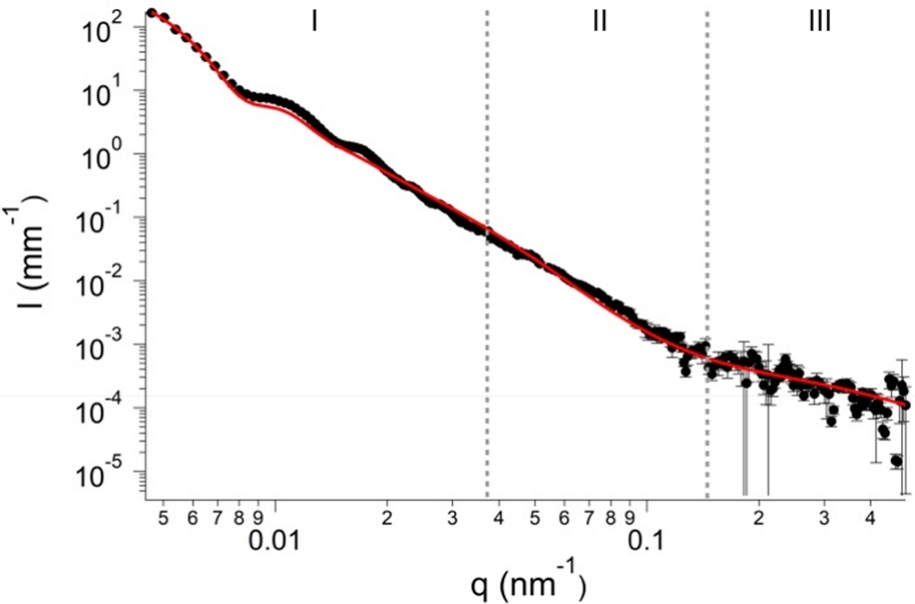
USAXS scattering profile for a dispersion of *S. epidermidis* at pH 7. Markers represent the data, and the red line represents the best fit according to the model proposed in Section 2.5.

Figure 2 shows the USAXS/SAXS scattering profile for a dispersion of *S. epidermidis* at pH 7. Here we can identify three main regions for this Gram-positive bacterium. At the lowest *q*, region I represents the largest scale in our system: The overall size of the bacterial cells including information on the core–shell structure, visible as oscillations, can be accessed. Effects on the thickness of cell wall and surface are contained in region II, while region III shows the structural arrangement of the population of objects dispersed in the cytoplasm, such as DNA, ribosomes, and proteins.

The parameters used to fit the data, as the scattering length densities of the bacterial cell core, peptidoglycan layer, and buffer solution, were extracted from Semeraro et al.,^12^ where the ultrastructure of *E. coli* cells was resolved by small angle scattering techniques. As we proposed a simplified model, the SLD of what is considered the bacterial wall had to be calculated considering the average value of inner membrane, periplasmic space, and the peptidoglycan contribution and then allowed to vary around this value. From the fitting shown in Figure 2, we obtained a cell radius of 419 ± 22 nm and a wall thickness of 41 ± 15 nm. For the cytoplasm content, we found a radius of gyration of 5 nm, which is a good average among the sizes of DNA, proteins, and ribosomes.^6^

**Figure 3:**
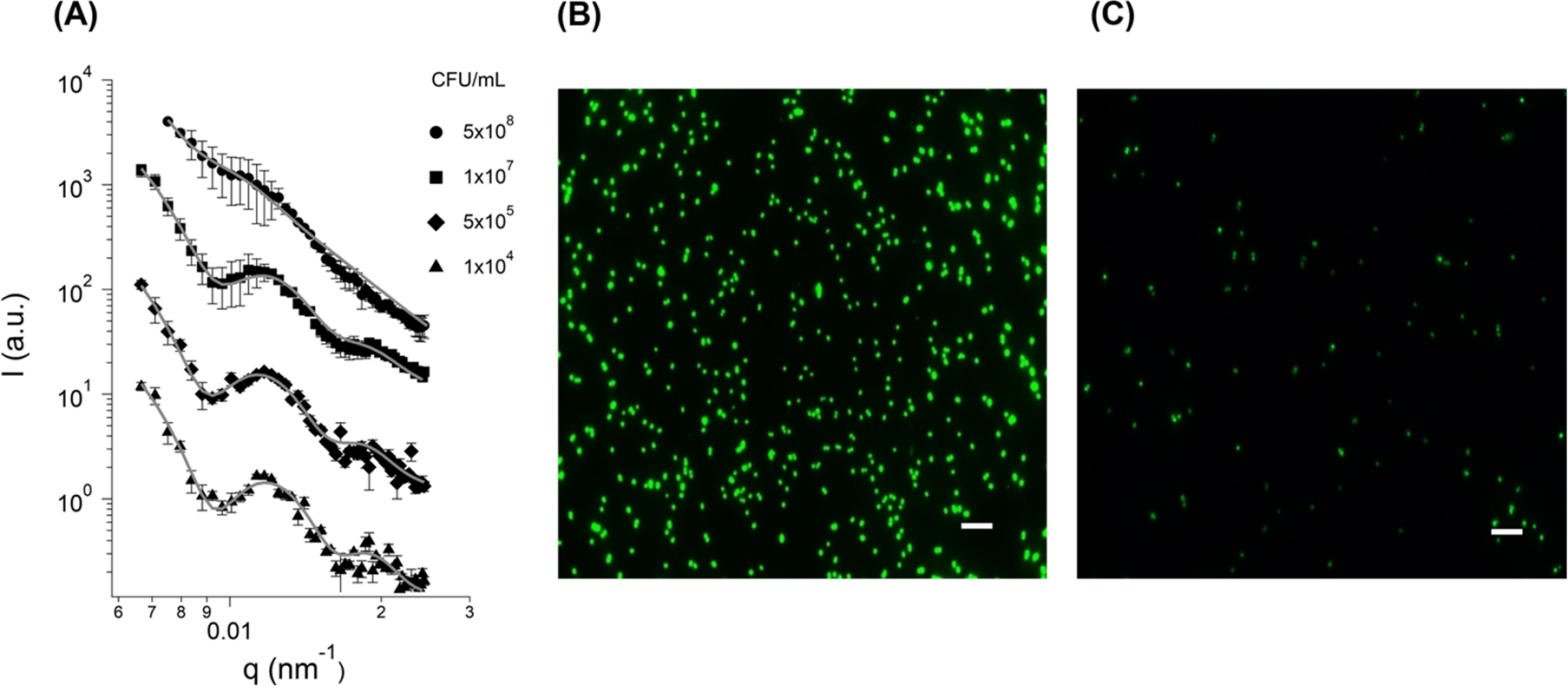
(A) Core–shell model fittings (lines) applied to static light scattering profiles (dots) obtained for a range of concentrations of cells dispersed in water. Fluorescence microscopy micrograph of a cell dispersion containing (B) 10^8^ CFU/mL and (C) 10^7^ CFU/mL.

Synchrotron experiments require quite some time to be prepared and strongly depend on the availability of the facility. Comparing USAXS data with light scattering can provide useful information, allowing this and similar methods to be more accessible to research. In the set of the first SLS experiments, an adequate concentration of bacteria had to be chosen. For this, a set of dilutions of an initial bacterial dispersion was prepared. As can be seen in Figure 3A, the light scattering profile is concentration dependent. At the highest concentration (5 × 10^8^ CFU/mL), a smearing of the form factor occurs due to cell aggregation. The aggregation is clearly evidenced when comparing fluorescence micrographs shown in Figure 3B,C for the 5 × 10^8^ CFU/mL and 10^7^ CFU/mL samples. Lowering the bacterial cell concentration reduces the probability of cell aggregates, improving the scattering pattern and consequently the form factor reliability. Concentration effects on the structural parameters extracted from SLS curves are reported in Table S2 confirming that 10^7^ CFU/mL is the highest concentration where the aggregation does not significantly affect the form factor. Below this concentration, it becomes quite difficult to observe bacterial cells by fluorescence microscopy. Since a compromise had to be found among acquisition time, data quality (Table S1), and right cell concentration for all techniques used, we decided to work with samples at about 1 × 10^7^ CFU/mL.^28^

A core–shell fitting was applied to the SLS data at this concentration, and an average radius of 358 ± 18 nm and a wall thickness of 34 ± 4 nm for *S. epidermidis* were obtained. Though a smaller radius and wall thickness were obtained from SLS, it is still in good agreement with what is reported in this work and by Wyatt.^8,9^

**Figure 4:**
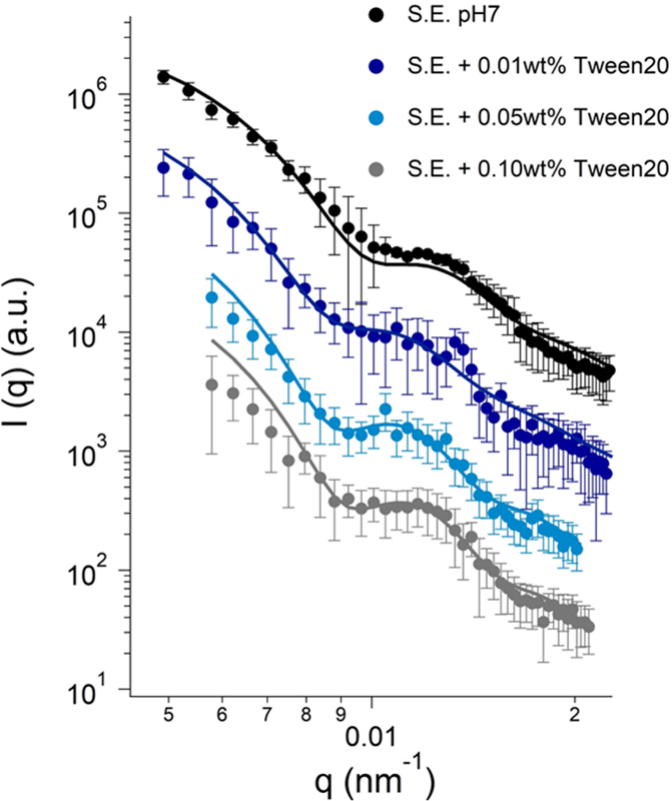
Light scattering profiles for *S. epidermidis* cells dispersed in water after exposure to 0.01, 0.05, and 0.1 wt % of Tween 20 and correspondent spherical core–shell model fitting. For the sake of clarity, data and respective fittings curves were shifted along the *y*-axis.

To make sure that the method is also applicable in determining variations of the overall structure and the thickness of the cell wall, the effect of Tween 20 was tested. This nonionic surfactant is well-known to disrupt the cell wall in bacteria.^29^Figure 4 shows the scattering curves of *S. epidermidis* cells exposed to increasing concentrations of Tween 20 up to 0.1 wt % along with fits using the core–shell model (see Table S3 for best fitting parameters). The characteristic oscillations in the scattering curves shift toward low *q* as the concentration of Tween 20 is increased. Two main effects result from the increase in concentration of Tween 20: a progressive reduction in the average wall thickness from 34 ± 4 to 16 ± 1 nm and an increase in the inner radius from 340 ± 2 to 430 ± 5 nm for the untreated sample and for the 0.1 wt % Tween 20 case, respectively. According to the presented data, the effect of Tween 20 on the bacteria is quantifiable in a gradual reduction of the membrane thickness accompanied by a swelling of the cytoplasmatic core.

### 3.2 pH Effect

**Figure 5:**
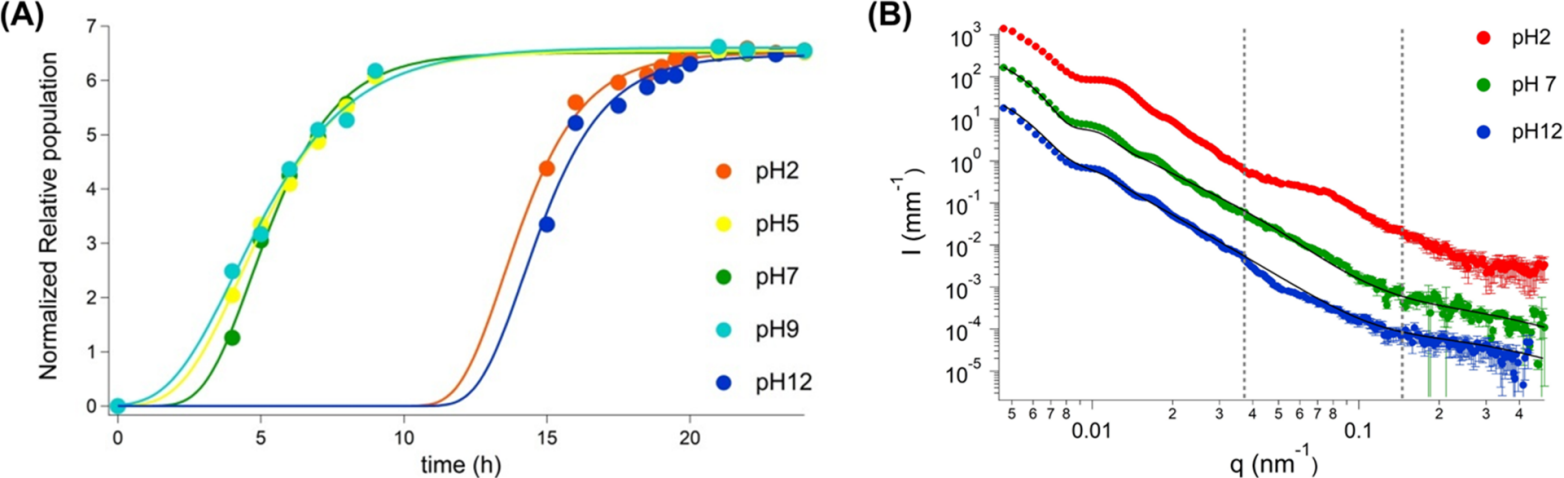
(A) Example of Gompertz model fitting a set of growth data for *S. epidermidis* cells previously exposed for 1 h to PBS at different pH values from 2 to 12. (B) USAXS/SAXS scattering profile of *S. epidermidis* cells at pH 2 (red markers), 7 (green markers), and 12 (blue markers) and fits (continuous black line) obtained for pH 7 and 12.

pH is known to affect bacterial growth and reduce the number of viable units up to a certain extent.^30^ When at an extreme pH, many micro-organisms and enzymes will cease their activity. Different components of the cell wall can be protonated (low pH) or deprotonated (high pH), inducing various bacterial responses.^31−35^ The lag phase time was used as an indicator for the reduction in CFU/mL caused by a change in pH. This parameter was obtained by fitting *S. epidermidis* growth curves for different pH environments by using the Gompertz model (see Figure 5A). At pH values close to 7 (i.e., 5 and 9), the bacterial growth is almost unaffected. When exposed to extreme pH values (i.e., 2 and 12), the onset of the bacterial growth passes from a few hours to more than 12 h showing that the metabolism of the microbe takes time to adapt/respond to the new environment. Figure 5B shows the USAXS/SAXS scattering profiles obtained from *S. epidermidis* cells at these extreme pH values as compared to neutral pH. The USAXS scattering pattern from *S. epidermidis* cells exposed to pH = 12 is quite similar to the pH = 7 case. It seems that the main region affected was the one linked to the wall thickness and surface along with a slight reduction in the SLD of the bacterial wall, passing from 10.8 to 10.7. This is also clear considering the fitting results (see Table S4). When exposed to pH = 2, the scattering pattern from the bacterial cells presents reduced oscillations, and a new feature appears at about 0.08 nm^–1^, resembling a very broad interaction peak or a new population. Unfortunately, it was not possible to find a proper fit for this case. This could happen due to the presence of new populations with diverse scattering characteristics and to the extra charge induced by the protonation. Dead cells, leaked material, and cells which are still alive but with a reduced metabolism to survive the imposed stress must be considered. All of these different objects could, in principle, present a distinct form factor, while the protonation could generate an interaction potential, and the correspondent structure factor should be taken into account.

### 3.3 Hydrogen Peroxide Effect

**Figure 6:**
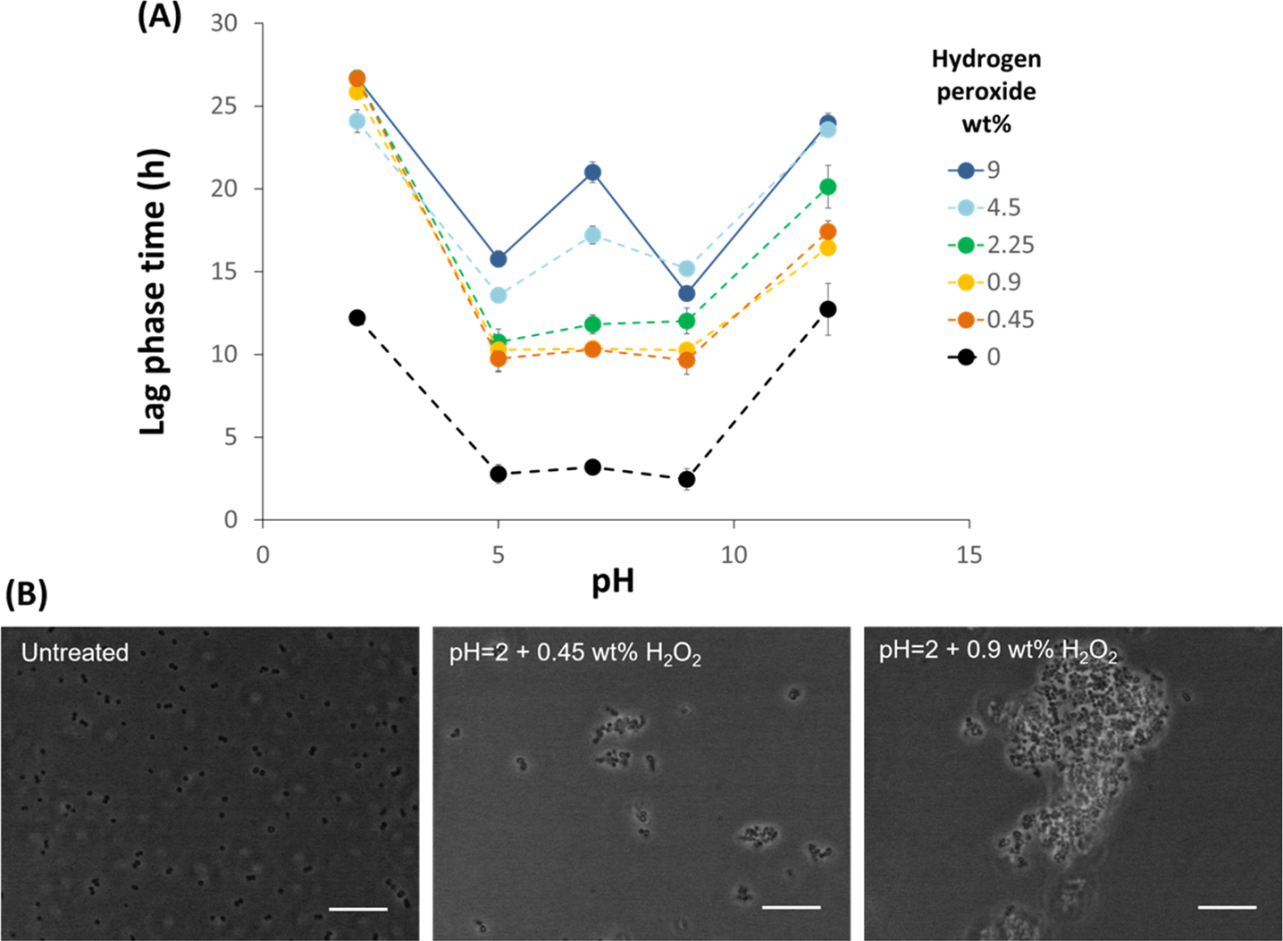
(A) Initial lag phase time after treatment with PBS at different pH values with and without hydrogen peroxide. (B) Polarized light micrograph of *S. epidermidis* cells dispersed in PBS (left to right): untreated, pH = 7, pH = 2 and 0.45 wt % of hydrogen peroxide and, pH = 2 and 9 wt % of hydrogen peroxide. Scale bar corresponds to 10 μm.

Hydrogen peroxide causes a progressive decrease of the viable CFU with increasing concentration. *S. epidermidis* is a catalase-positive bacterium, expressing this protein when in the presence of hydrogen peroxide which also causes a determinant change in the cellular environment.^36,37^Figure 6A shows the combined effect of pH (from 2 to 12) and hydrogen peroxide (from 0 to 9 wt %) on the lag phase duration, extracted from the Gompertz model. Such an approach was chosen to understand if, besides a reduction of viable microorganisms, these would also be affected in a way that would compromise the bacteria adaptation to a new environment. Even if at pH 12 there is a lower reduction of CFU/mL, it seems that *S. epidermidis* takes a slightly longer time to adapt. At the extremes (2 and 12), it takes about 13–15 h until there is noticeable microbial growth. The slight increase in lag time of approximately 1 h for pH 5, 7, and 9 might be associated with extra time needed for cells to adapt to the media after being in PBS. When hydrogen peroxide is added, the lowest lag phase time difference between the extremes in peroxide concentration occurs at pH = 2 (∼2.6 h). At this low pH, the CFU reduction is less dependent from hydrogen peroxide concentration than at any other tested pH value. For example, when exposed to pH 12, the lag phase time difference between the lowest and highest peroxide concentration increases to 8 h. This might be due to a synergy between pH and oxidation effects as a low pH is known to promote higher membrane permeability, where cells can no longer maintain the basal cytoplasm’s pH.^30^ This synergy was also identified by Jackett et al.^38^ when studying the resistance of *Mycobacterium tuberculosis* strains to hydrogen peroxide and low pH, and a mechanistic hypothesis on the action of singlet oxygen species was postulated by Dahl et al.^39^ Moreover, cell aggregation was observed at pH 2, increasing in the presence of hydrogen peroxide which is also confirmed by electron microscopy on the bacterial strain investigated in this study (Figure 6B).

**Figure 7:**
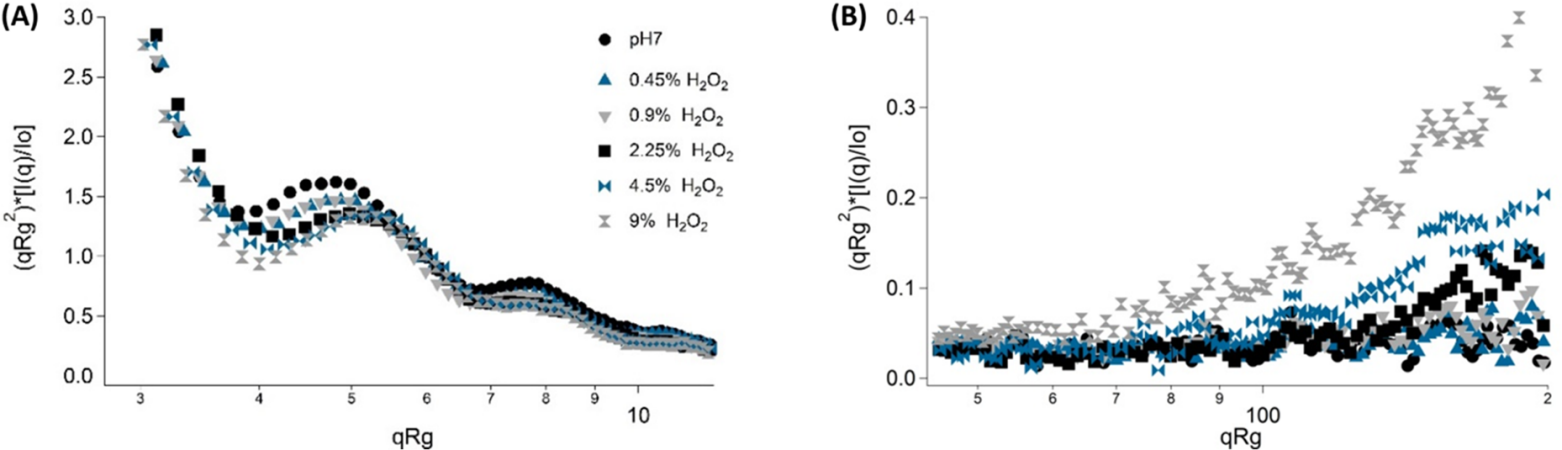
Dimensionless Kratky representation for *S. epidermidis* cells at pH 7 exposed to different hydrogen peroxide concentrations, zoom at low *q* (A) and high *q* (B). Legend of panel A applies also to panel B.

The triggering point for this aggregation process must be related to quorum sensing signals received by the bacterial population as individuals are being the target of an antimicrobial.^40,41^ When damaged by an antimicrobial agent, bacterial cells suffer structural changes as they impact the wall and the cytoplasm. The morphological changes can then be identified by small angle scattering techniques.^42−45^ Changes in the size of particles in solution can be detected by changes in the radius of gyration (*R*_g_) and in the forward scattering intensity (*I*(0)). *R*_g_ can be calculated from the slope of the linear region when the scattering data are in the Guinier representation (ln *I*(*q*) vs *q*^2^), and *I*(0) results from the associated intercept.^46^ The collected data does not have a *q* range extended enough to access the entire Guinier region^47^ (see Figure S5). The Guinier approximation can be considered valid when the extracted *R*_g_ meets the condition *qR*_g_ < 1.3, while in our case values of about 3 are found (i.e., the extracted values can be considered only as apparent). The obtained *R*_g_ values were then used to create dimensionless Kratky plots for a more detailed data evaluation (see Figure S6). Figure 7 shows magnifications of the dimensionless Kratky plot in the low *q* (panel A) and high q (panel B) regions.^48,49^ When looking to the low *q* region (see Figure 7A), the Kratky plot confirms that the scattering objects retain the globular shape even at the highest peroxide concentration and all curves are progressively shifted to higher *q*. This effect can be linked to a reduction in the size of the bacteria. The bumps are representative of a core–shell structure, and their shift to higher *q* along with the decrease in the intensity could be indicative of both a wall thickness reduction and concomitant destabilization of the peptidoglycan matrix. In the high *q* region (see Figure 7B), the increase in hydrogen peroxide concentration produces a proportional upturn in the scattering signal which is indicative of protein denaturation inside the cytoplasm due to the imposed stress. Overall, the identified differences in the scattering signal show that hydrogen peroxide promotes changes in different cellular regions of *S. epidermidis* cells.

**Figure 8:**
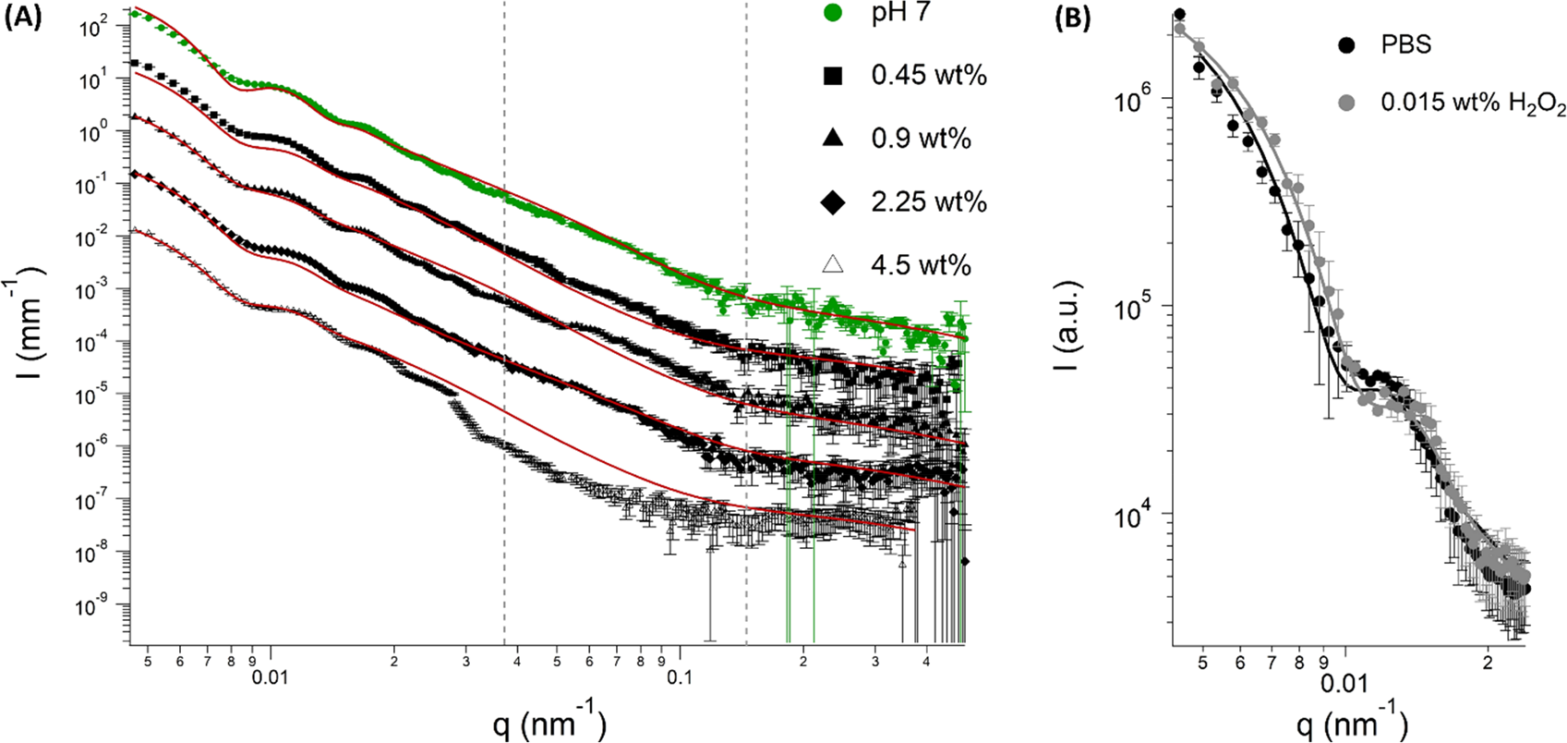
(A) USAXS/SAXS scattering profiles of *S. epidermidis* cells exposed to different concentrations of hydrogen peroxide at pH 7 and respective fits (dashed purple lines). In the presence of 4.5 wt % of hydrogen peroxide, it is worth noting the deviation from the fit in the region characteristic of the cell wall (3 × 10^–2^ < *q* < 1.5 × 10^–1^ nm^–1^). (B) Spherical core–shell model applied to light scattering profile obtained for *S. epidermidis* cells dispersed in water after exposure to PBS at pH 7 (black) and hydrogen peroxide (gray).

Figure 8 shows the effect of hydrogen peroxide on the USAXS/SAXS and SLS patterns at pH = 7. Data showed that the progressive increase in hydrogen peroxide promotes a decrease in the scattering intensity. This decrease is in good agreement with the reduction of *S. epidermidis* CFU. Applying the model proposed in Section 2.5 allowed for a more quantitative analysis with respect to bacteria size and wall thickness variations. Our quantitative results are in good agreement with qualitative considerations worked out by the Kratky plot approach, as there is a progressive reduction in bacteria size and polydispersity of all dimensions with an increasing hydrogen peroxide concentration. However it was only possible to properly fit the scattering data up to 4.5 wt % hydrogen peroxide because at 9.0 wt % the changes in scattering are such that it was not possible to apply the proposed model (Table S4). Even if Gram-positive bacteria lacks the outer cell membrane, SLD changes might also occur on the peptidoglycan. Antimicrobial agents as hydrogen peroxide might promote swelling of the less crystalline domains, changing the electron density and further permeating the bacterial wall. Ultimately, when reaching the cytoplasm, hydrogen peroxide could promote DNA and protein oxidation due to reactive oxygen species, which would alter the cytoplasm SLD and render the fitting difficult at very high H_2_O_2_ concentration. Figure 8B reports the SLS data and fittings for *S. epidermidis* cells dispersed in water after 1 h exposure to pH = 7 with and without hydrogen peroxide. When cells were exposed to hydrogen peroxide, changes in size and thickness were observed, with a slight decrease in size (330 ± 15 nm) and wall thickness (25 ± 5 nm). Such a difference would not be expected as the used hydrogen peroxide concentration is quite low when compared to bactericidal concentrations present in the literature.^20^ In this case, it is mainly expected to identify structural changes caused by cellular injuries due to hydrogen peroxide action, rather than more pronounced changes that could lead to cell death.^20^

## 4 Conclusions

In this work, synergy between pH and hydrogen peroxide is shown, which, in addition to a reduction in *S. epidermidis* CFU, also promotes an increase in the initial lag phase. This delay seems to be linked to the observed aggregation behavior of *S. epidermidis* cells when in contact with 9 wt % of hydrogen peroxide. Bacterial cells on the outside of the cluster should be most affected, and many can be dead. When dispersed back into the media, the viable cells surrounded by dead ones will need some time to reach the media. After a certain time, they adapt (end of lag phase) and enter the exponential phase. Modeling of scattering patterns of *S. epidermidis* cells obtained from USAXS/SAXS and SLS techniques was presented. We used a multiscale core–shell approach for *S. epidermidis* cells exposed to different environments and dispersed in PBS. The analysis was performed on the bacterial dispersion without any purification. Scattering patterns by X-rays and light provide evidence of structural changes on the Gram-positive bacteria strain caused by the action of hydrogen peroxide with particular regard to the overall size and cell wall thickness. Hydrogen peroxide and pH showed a synergistic action. Increasing concentrations of hydrogen peroxide promoted a reduction in cell wall thickness which can be mainly linked to the oxidation of the peptidoglycan layer.^20,39^ This effect was also noticed when exposing *S. epidermidis* cells to Tween 20, where increasing concentrations of the surfactant led to a decrease in cell wall thickness with a concomitant increase in the overall bacteria size as a result of a prominent swelling of the cytoplasm.^50^ The combined USAXS/SAXS and SLS approach allows for a quantification of the structural changes of the wall, opening the use of these laboratory scale methods as a tool to screen new active molecules and mixtures for a selective bacteria wall disruption. This approach will now be used to perform further investigations regarding other classes of surfactants and create more complex formulations with the objective of idenifying synergies and disclosing a mechanistic understanding of the antimicrobial mechanisms involved.

## Supporting Information Available

Brief description of the serial dilution method and Gompertz model application to different inoculum concentration, time to detection method applied to dilution series, Guinier representation for *S. epidermidis* cells at pH 7, dimensionless Kratky representation for *S. epidermidis* cells at pH 7 exposed to different hydrogen peroxide concentrations, comparison of parameters used for modeling *S. epidermidis* cells in this work and *E. coli* in Semeraro et al.,^12^ and inner cell radius and wall thickness values obtained from fitting of SLS and USAXS/SAXS data. (PDF)
