# Supplementary material for: Ultra-/Small Angle X-ray Scattering (USAXS/SAXS) and Static Light Scattering (SLS) Modeling as a Tool to Determine Structural Changes and Effect on Growth in S. epidermidis
Source: ACS Appl Bio Mater. 2022 Jul 29;5(8):3703–12. doi: 10.1021/acsabm.2c00218 (PMC9940853; doi:10.1021/acsabm.2c00218)
Supplement: Supplementary file 1 — mt2c00218_si_001.pdf [file mt2c00218_si_001.pdf]

SUPPLEMENTARY INFORMATION

USAXS/SAXS and SLS modelling as a tool to determine structural changes and effect on growth in S. epidermidis

Hugo Duarte[1,#,*], Jeremie Gummel[2], Eric Robles[3], Debora Berti[1], Emiliano Fratini[1,*]

[1]Department of Chemistry "Ugo Schiff" and CSGI, University of Florence, Sesto Fiorentino, Florence I-50019, Italy

[2]Brussels Innovation Centre, Temselaan 100 Strombeek-bever, B-1853 Belgium

[3]Household Care Analytical, Procter & Gamble Newcastle Innovation Centre, Newcastle NE12 9TS, United Kingdom

[*]Corresponding author: hmduarte@ualg.pt, emiliano.fratini@unifi.it

[#]Current address: MED – Mediterranean Institute for Agriculture, Environment and Development, Universidade do Algarve, Faculdade de Ciências e Tecnologia, Campus de Gambelas, Ed. 8, 8005-139 Faro, Portugal

Modelling bacterial growth – Modified Gompertz model explanation and validation

Serial dilution method was applied as schematized in figure S1. As a first step, S. Epidermidis cells' growth on TSB was assessed as can be seen in figure S2. The growth curve shown is the result of a set of experiments where three different inoculum concentrations were tested with good reproducibility and accuracy as in the lag phase the measured optical density at 600 nm was 0.317±0.002. After, optical density values were correlated with the colony forming units per volume (CFU/mL) by the serial dilution method[1,2]. While cells were growing, aliquots were taken at certain times, a series of dilutions were prepared and then plated as shown.

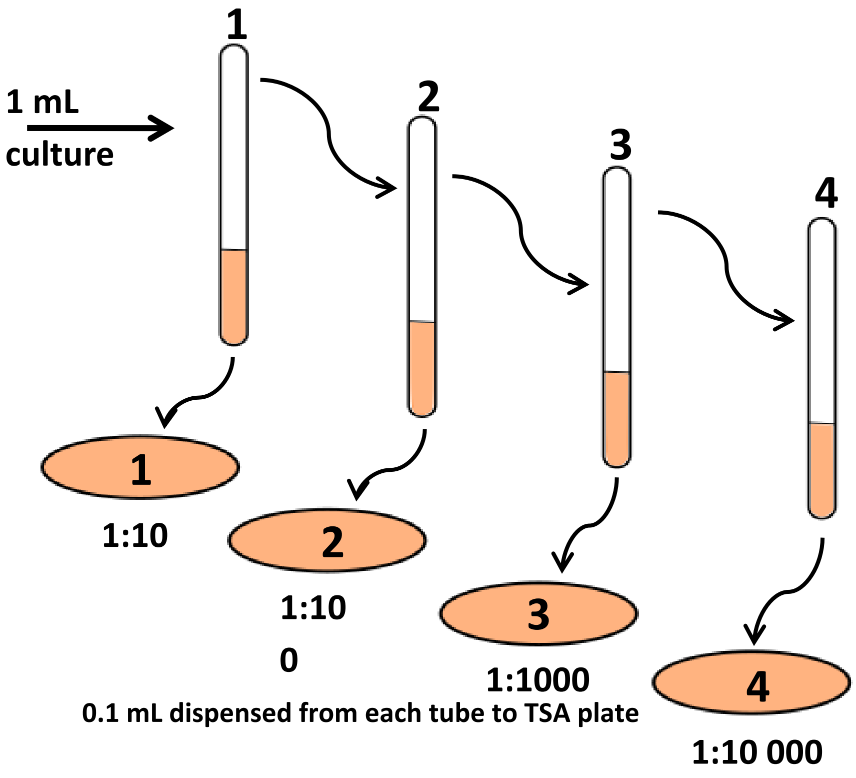

Figure S1 – Serial dilution method, adapted from [1].

Then, a suitable model to fit the growth curve was chosen and Gompertz function[3,4] was proven to be in good agreement with the data from which the maximum specific growth rate (μ_{max}) and lag phase (λ) can be extracted. The modified Gompertz model is described as follows:

$$y = Aexp\left\{-exp\left[\frac{\mu_m \cdot e}{A}(\lambda - t) + 1\right]\right\} \qquad \textbf{(S1)}$$

As bacteria grow exponentially it is common to plot the logarithm of the relative population size, $y = ln(N/N_0)$, as a function of time, t. Considering three phases of growth the parameters are described: the plateau, $A = ln(N_\infty/N_0)$, is the maximum value reached, the maximum specific growth rate, μ_m, defined as the tangent at the inflection point and finally the lag time, λ, which is the x-axis intercept of the overmentioned tangent and e represents the Neper number[3].

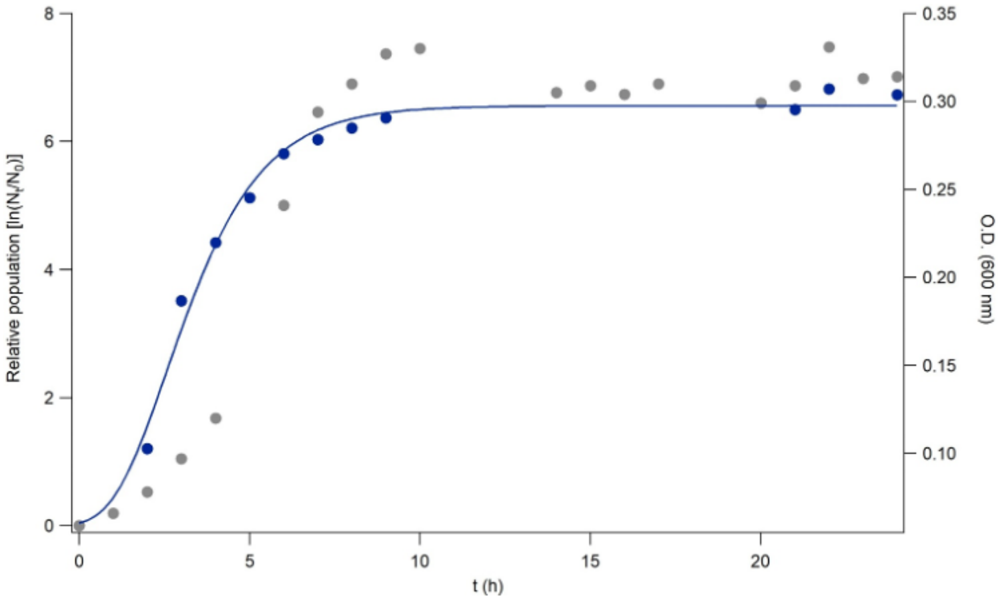

Figure S2 – *S. Epidermidis* growth curve (grey markers) and Gompertz model fitting applied to relative population (blue markers are the data and the line is the fitting).

In order to define any deviation on the parameters obtained using the Gompertz function, dilutions of the inoculum were prepared (see figure S3), Gompertz model was applied and the time-to-detection (TTD) method according to the one described by Baka et al.[5] This has been done as the lag phase is the parameter of interest in this study and its estimation is quite sensitive, much more than the growth rate. In our case, the difference between the lag phase extracted from Gompertz model ranges from approximately 1.0±0.2 h to 1.5±0.2 h by TTD (y = 0.625x + 1.466 where the slope is the maximum specific growth rate, and the intersection is the lag phase) (see figure S4) which can be negligible as will be shown in the results section since we worked in a longer time range. The main

relevance given to the lag phase is due to the approach taken, similarly as described Baldry et al.[6] where bacteriostatic and bactericidal tests were performed. Using this approach, we intended to understand if besides reducing the number of viable cells how the ones still alive could eventually be affected and how would these respond when again inoculated in fresh media.

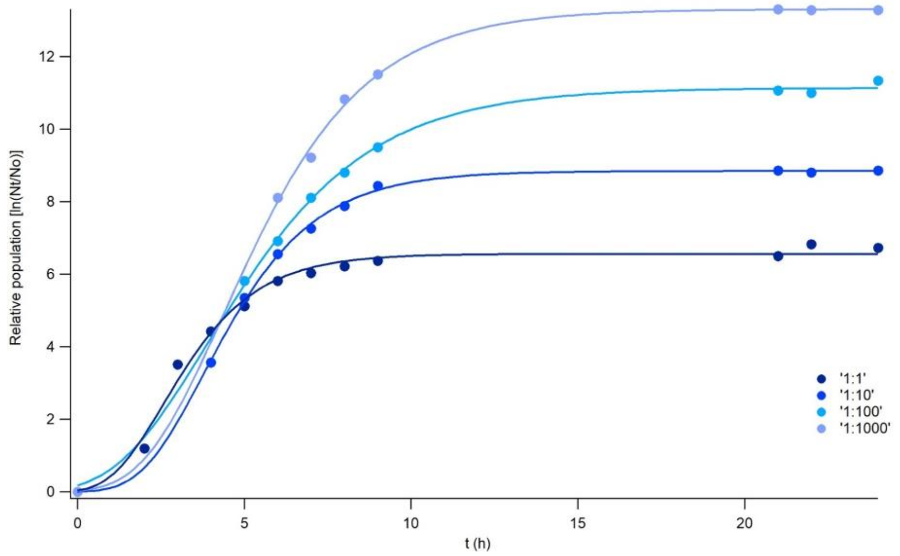

Figure S3 – Gompertz model fittings applied to growth curves obtained from different inoculum concentrations.

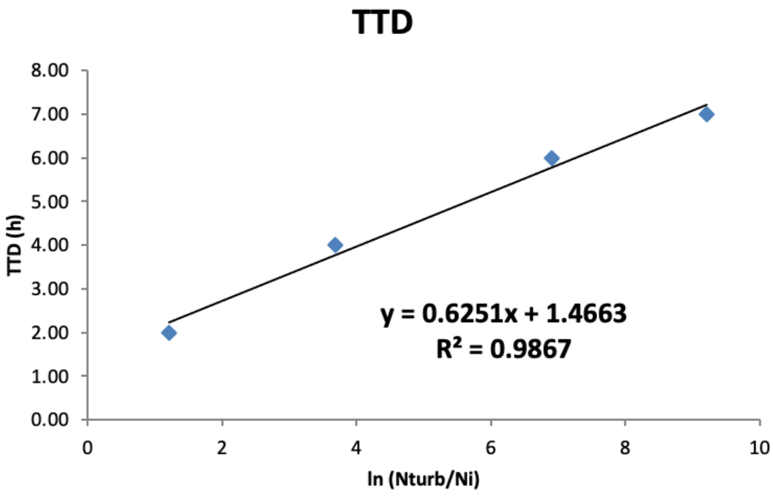

Figure S4 – Time-to-detection method applied to dilutions shown in figure S1.

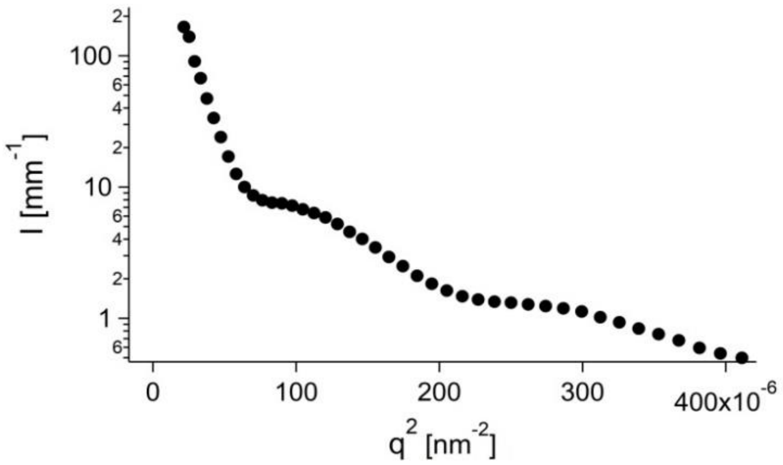

Figure S5 – Guinier representation for *S. epidermidis* cells at pH 7 USAXS/SAXS data.

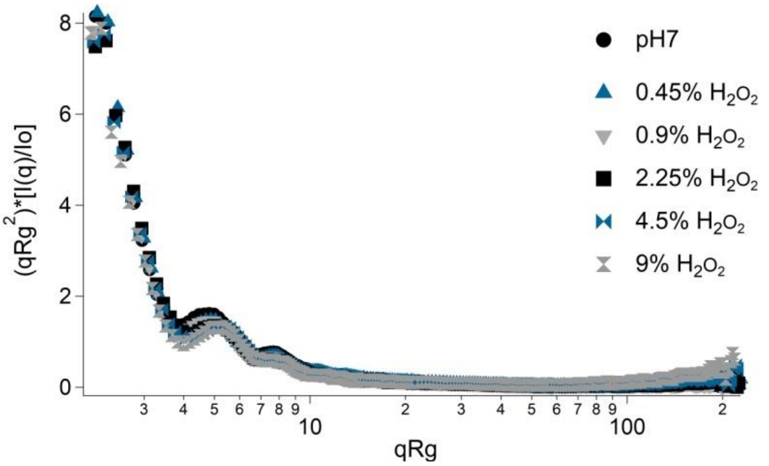

Figure S6 – Dimensionless Kratky representation for S. Epidermidis cells at pH 7 exposed to different hydrogen peroxide concentrations.

Table S1 – Average SLD values used by Semeraro et al.[7] and in this work.

Parameter	Semeraro et al.[7]	This work
Average SLD of the cytoplasm	10.26	10.26
Average SLD of the peptidoglycan layer	11.64	10.80
SLD of the buffer solution	9.47	9.47

Table S2 – Fitting results for inner cell radius and wall thickness and correspondent polydispersity from SLS data, depending on CFU/mL**.**

CFU/mL	$5x10^8$	$1x10^7$	$5x10^5$	$1x10^4$
r_c (nm)	515±155	358±18	360±7	368±6
Thickness (nm)	21±79	34±4	39±4	35±3
poly(r_c)	0.26±0.2	0.23±0.02	0.12±0.03	0.12±0.03
poly(thickness)	0.1±0.9	0.56±0.04	0.99±0.9	0.99±0.9
X^2	8.27	1.64	2.15	4.55

Table S3 – Inner cell radius, thickness and polydispersity (poly) of radius and thickness extracted from the core-shell model fitting applied to light scattering data obtained from dispersion of cells previously exposed to Tween 20.

Tween 20 (wt %)	0	0.01	0.05	0.1
r_c (nm)	358±18	402±4	436±4	430±5
thickness (nm)	34±4	33±1	23±1	16±1
poly(r_c)	0.23±0.02	0.21±0.02	0.16±0.02	0.15±0.01
poly(thickness)	0.56±0.04	0.10±0.01	0.05±0.01	0.04±0.01
X^2	1.64	0.28	0.31	0.69

Table S4 – Fitting results for cell radius and wall thickness and correspondent polydispersity from USAXS/SAXS curves.

			pH7 + H_2O_2 (wt%)			
	pH7	pH12	0.45	0.9	2.25	4.5
r_c (nm)	419±22	408±9	392±34	381±16	395±25	355±42
poly(r_c)	0.20±0.05	0.22±0.01	0.21±0.07	0.25±0.06	0.28±0.06	0.21±0.10
thickness (nm)	41±15	42±6	50±11	49±14	22±35	68±56
poly(thickness)	0.81±0.01	0.99±0.01	0.94±0.01	0.81±0.01	0.57±0.01	0.81±0.01
X^2	0.86	0.62	0.67	0.64	0.65	2.1

References

1. Ben-David, A. & Davidson, C. E. Estimation method for serial dilution experiments. *J. Microbiol. Methods* **107**, 214–221 (2014).

2. Thomas, P.; Sekhar, A. C.; Upreti, R.; Mujawar, M. M. & Pasha, S. S. Optimization of single plate-serial dilution spotting (SP-SDS) with sample anchoring as an assured method for bacterial and yeast cfu enumeration and single colony isolation from diverse samples. *Biotechnol. Reports* **8**, 45–55 (2015).

3. Zwietering, M. H.; Jongenburger, I.; Rombouts, F. M. & van 't Riet, K. Modeling of the Bacterial Growth Curve. *Appl. Environ. Microbiol.* **56**, 1875–1881 (1990).

4. Li, B.; Qiu, Y.; Shi, H. & Yin, H. The importance of lag time extension in determining bacterial resistance to antibiotics. *Analyst* **141**, 3059–3067 (2016).

5. Baka, M.; Noriega, E.; Stamati, I.; Logist, F. & Van Impe, J. F. M. Critical assessment of the time-to-detection method for accurate estimation of microbial growth parameters. *J. Food Saf.* **35**, 179–192 (2015).

6. Baldry, M. G. C. The bactericidal, fungicidal and sporicidal properties of hydrogen peroxide and peracetic acid. *J. Appl. Bacteriol.* **54**, 417–423 (1983).

7. Semeraro, E. F.; Devos, J. M.; Porcar, L.; Forsythb, V. T. & Narayanan, T. In vivo analysis of the Escherichia coli ultrastructure by small-angle scattering. *IUCrJ* **4**, 751–757 (2017).
